# A Lightweight Modified Adaptive UNet for Nucleus Segmentation

**DOI:** 10.3390/s26020665

**Published:** 2026-01-19

**Authors:** Md Rahat Kader Khan, Tamador Mohaidat, Kasem Khalil

**Affiliations:** 1 Electrical and Computer Engineering Department, University of Mississippi, Oxford, MS 38677, USA; mkaderk@go.olemiss.edu (M.R.K.K.); tnmohaid@go.olemiss.edu (T.M.); 2 Department of Electrical Engineering, Assiut University, Assiut 71515, Egypt

**Keywords:** deep learning, nucleus segmentation, UNet, image segmentation

## Abstract

Cell nucleus segmentation in microscopy images is an initial step in the quantitative analysis of imaging data, which is crucial for diverse biological and biomedical applications. While traditional machine learning methodologies have demonstrated limitations, recent advances in U-Net models have yielded promising improvements. However, it is noteworthy that these models perform well on balanced datasets, where the ratio of background to foreground pixels is equal. Within the realm of microscopy image segmentation, state-of-the-art models often encounter challenges in accurately predicting small foreground entities such as nuclei. Moreover, the majority of these models exhibit large parameter sizes, predisposing them to overfitting issues. To overcome these challenges, this study introduces a novel architecture, called mA-UNet, designed to excel in predicting small foreground elements. Additionally, a data preprocessing strategy inspired by road segmentation approaches is employed to address dataset imbalance issues. The experimental results show that the MIoU score attained by the mA-UNet model stands at 95.50%, surpassing the nearest competitor, UNet++, on the 2018 Data Science Bowl dataset. Ultimately, our proposed methodology surpasses all other state-of-the-art models in terms of both quantitative and qualitative evaluations. The mA-UNet model is also implemented in VHDL on the Zynq UltraScale+ FPGA, demonstrating its ability to perform complex computations with minimal hardware resources, as well as its efficiency and scalability on advanced FPGA platforms.

## 1. Introduction

Medical image segmentation is a crucial process in medical imaging that involves mapping and extracting anatomical features or regions of interest from complex images. This plays a fundamental role in various clinical applications, including disease diagnosis, treatment planning, and surgical navigation [1,2,3,4,5,6]. It simplifies quantitative analysis by effectively partitioning images into meaningful components, enabling specialists to make more informed decisions and improve patient outcomes. With advances in computational methods such as machine learning, deep learning, and computer vision, medical image segmentation continues to evolve, yielding increasingly precise and efficient solutions for image analysis and interpretation [7,8,9,10]. Specifically, microscopy image segmentation is essential for extracting valuable information from microscopic images, aiding in tasks such as cell analysis, tissue characterization, and disease diagnosis [11,12]. Despite recent advancements, challenges such as dataset variability and class imbalance persist, underscoring the need for innovative segmentation techniques [13,14].

Nucleus segmentation, a critical task in biomedical image analysis, involves identifying and outlining cell nuclei within microscopic images. Accurate nucleus segmentation is essential for understanding cellular processes, diagnosing diseases like cancer, and facilitating quantitative analysis for researchers and clinicians [15,16,17]. However, despite progress in imaging technologies and computational methods, nucleus segmentation remains challenging due to factors such as variability in nuclear morphology, image noise, and overlapping structures, emphasizing the need for continued research and development in this subject.

Convolutional Neural Networks (CNNs) are a well-established subset of image segmentation models that have become a cornerstone of the field, demonstrating exceptional performance across various tasks [18,19,20,21,22,23]. Among these models, U-Net [24] stands out as a specialized CNN architecture for biomedical image segmentation [25,26], praised for its efficiency and adaptability. Its U-shaped architecture, which includes an expanding path for precise localization and a contracting path for feature extraction, enables successful segmentation even with limited training data. U-Net’s skip connections allow for the smooth integration of high-level and low-level information, improving segmentation performance, particularly when dealing with complex anatomical structures or limited target regions. However, convolutional algorithms often struggle to capture long-range dependencies and global context in images due to the inductive biases inherent in convolutional operations, limiting improvements in segmentation accuracy. Recent research has proposed Generative Adversarial Networks (GANs) [27] and self-attention mechanisms [5] to address these limitations. Additionally, enhancements to the U-Net architecture, such as UNet++ [28], improve feature representation and contextual information through nested skip pathways, dense skip connections, and deep supervision mechanisms, significantly enhancing segmentation performance in scenarios with complex structures or small objects [29].

Transformers [12], originally developed for natural language processing, have been adapted for various computer vision tasks, including medical image segmentation. Transformers capture contextual information for segmentation by dividing images into patches and applying self-attention mechanisms [7]. They can also be combined with encoder–decoder architectures, where the encoder extracts features from image patches, and the decoder generates segmentation masks. The transformer architecture offers advantages such as parallel processing, attention mechanisms, and scalability, making it particularly suited for large-scale medical imaging datasets.

Oktay et al. [30] introduced Attention U-Net, a model designed for pancreas segmentation in medical images. The incorporation of attention mechanisms into the U-Net architecture revolutionizes segmentation by enabling the model to selectively focus on critical regions, resulting in significant performance gains. This approach addresses the challenge of identifying the pancreas in complex anatomical contexts with varying image quality. Jha et al. [31] proposed DoubleU-Net, which integrates two parallel U-Net structures to capture multi-scale features. This architecture enhances segmentation accuracy by capturing both local and global contextual information, particularly in regions with complex structures or class imbalance. Tomar et al. [32] presented FANet, a novel Feedback Attention Network that dynamically refines feature representations through feedback and attention mechanisms. FANet effectively integrates these techniques to capture fine details in biomedical imaging, improving segmentation accuracy. Lin et al. [33] introduced the DS-TransUNet architecture, which uses dual Swin transformer blocks to enhance feature extraction and representation learning while maintaining the spatial feature integration of U-Net. By leveraging the strengths of convolutional and transformer-based models, this hybrid architecture effectively addresses the complex challenges associated with medical image segmentation. Zhang et al. [34] proposed the MTC-TransUNet architecture, which merges the strengths of transformer networks with the efficiency of U-Net for image segmentation. By integrating multi-scale mixed convolutions into the TransUNet architecture, the model captures features at different scales, improving its ability to accurately segment medical images.

However, previously proposed methods focused on various tasks where the ratio of background and foreground pixels was ignored. As a result, those methods perform poorly on imbalanced datasets, like nucleus segmentation. Additionally, these large models often overfit the dataset due to their large parameter sizes, resulting in reduced robustness. In this work, we propose a novel framework for nucleus segmentation that addresses those issues. Our framework comprises a newly developed architecture, mA-UNet, a lightweight, modified adaptive UNet model, and a data preprocessing approach, the Adaptive Augmentation (ADA) algorithm [35], which was originally proposed for a different domain. The following is a summary of our contribution:

- We develop a new light-weight adaptive UNet architecture called mA-UNet, which specializes in predicting small foreground objects like a nucleus.
- The proposed framework uses an ADA module [35], an enhanced augmentation method that extracts patches using unit kernel convolution, leading to a more complete semantic representation of an imbalanced dataset and a faster learning rate for the model.
- Both qualitative and quantitative studies show superior results compared to other previously proposed architectures.
- Due to its lightweight nature and reduced parameter count compared to other cutting-edge models, the proposed architecture possesses the capability to retain information efficiently. Consequently, it takes shorter training times, rendering it a dependable solution for automated medical image segmentation in real-world applications.
- The mA-UNet model is implemented on the Zynq Ultra-Scale+ using VHDL, demonstrating its suitability for high-performance applications on advanced FPGA architectures.

The rest of the paper is structured as follows: Section 2 explains the details of our proposed framework; Section 3 describes the implemented dataset, training scheme and performance evaluation; and the conclusion and future work are outlined in Section 4.

## 2. The Proposed Method

**Figure 1:**
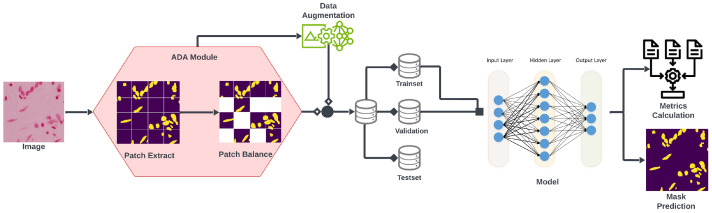
The overall strategy of the proposed framework involves initial passage of images through the ADA module, followed by dataset creation through integration with data augmentation, while simultaneously performing mask prediction and evaluation metric computation (including MIoU, F-1 score, precision & recall).

In this study, we present an innovative framework aimed at improving nucleus segmentation by addressing previous limitations. Our comprehensive framework comprises a novel architecture, mA-UNet, characterized by its lightweightness and adaptability, and a data preprocessing strategy, the ADA algorithm [35]. Initially, the dataset undergoes processing through the ADA module [35], generating balanced patch images, with a subset further subjected to basic data augmentation techniques such as vertical flip, horizontal flip, random rotation, and blur. Subsequently, the entire dataset is partitioned into three subsets: training, validation, and testing, in accordance with an 80:10:10 ratio. The workflow of our framework is illustrated in Figure 1, describing its functionality and components.

### 2.1. Adaptive Augmentation

The ADA module [35], leveraging insights from CNN architectures, introduces an advanced augmentation methodology characterized by the extraction of multi-receptive features in the form of patches through unit kernel convolution across diverse scale levels. This approach results in a more comprehensive semantic representation and a faster learning rate for the model [36]. In operational terms, this algorithm systematically isolates patches of a predetermined magnitude from each image encompassed within the dataset. A critical analysis of existing patch libraries underscores their inherent limitations, notably the absence of provisions ensuring class equilibrium and the inability to foster feature overlap, compounded by their inadequacy in accommodating disparate input configurations. However, the ADA algorithm [35] pioneers the integration of a recently formulated mathematical formulation, markedly enhancing augmentation efficiency. This innovation augurs well for its applicability across a broad spectrum of variable input geometries, further underscoring its significance in the domain of image processing and machine learning.

(1) $$p_{h}=\left\lceil \frac{h-k}{s}\right\rceil +1$$

(2) $$p_{l}=\left\lceil \frac{w-k}{s}\right\rceil +1$$

(3) $$N=p_{h}\times p_{l}$$

(4) $$N_{\alpha }=p_{h}\times p_{l}-\alpha$$

In this context, the application of pixel-wise convolution to the input image initiates the formation of patches, with a padding value, denoted as *p*, set to 0. The incorporation of the dilation parameter, *d*, emerges as a strategic mechanism to streamline the architectural intricacies of CNN-based networks. Moreover, the distortion potential of patches arises due to the utilization of pixel-wise convolution within the ADA module [35], facilitating their extraction from images preparatory to CNN-based architecture training. Additionally, in order to uphold class equilibrium, an algorithmic component termed the Improper Class Distribution (ICD) factor, denoted as α in Equation (4), is introduced. Here, α signifies the extent of patch mitigation subsequent to employing the ADA approach [35]. Parameters $p_{h}$ and $p_{l}$ denote the horizontal and vertical movements of the unit kernel, respectively, contingent upon the image’s height, *h*, width, *w*, patch size, *p*, and stride, *s*. Notably, *N* and $N_{\alpha }$ symbolize the patch quantities generated from images devoid of and subject to alpha correction, respectively. Equation $N_{\alpha }$ is enlisted within the ADA algorithm [35] framework to preserve optimal class distribution throughout the training regimen.

### 2.2. Proposed mA-UNet

In this section, we present a comprehensive explanation of our novel mA-UNet framework tailored specifically for nucleus segmentation. At its core lies a UNet [24] architecture serving as the foundational scaffold. This U-shaped framework, comprising both encoder and decoder components, processes input images, generating diverse feature maps that subsequently pass through crafted bridge layers. The culmination of this journey yields the ultimate prediction output. Notably, our encoder–decoder design consciously eschews the utilization of pre-trained model weights, thereby affirming the model’s autonomy. However, the incorporation of the ADA augmentation module [35] significantly improves the overall performance of our model. A comprehensive breakdown of the entire network configuration is described in Table 1. The table enumerates crucial parameters such as kernel width, kernel height, and kernel count for each convolutional layer, alongside the resultant output sizes of the feature maps. Across all layers, with the exception of the output layer, Rectified Linear Unit (ReLU) activation function [37] is deployed, while the output layer employs Softmax activation.

The model architecture incorporates two distinctive components: a downblock responsible for downsampling and an upblock for upsampling. Initially, the downblock-1 and upblock-1 consist of convolutional networks with a kernel size of two (3×3), maintaining identical padding dimensions. After convolution, each layer is activated by a ReLU activation function, followed by a dropout layer to enhance model robustness. Downblock-2 through downblock-6 adhere to a similar structural pattern as downblock-1, although augmented with a 2×2 max-pooling layer to halve the feature height and width dimensions. Upblock-1 mirrors the configuration of downblock-1 with the inclusion of a 2×2 convolutional layer following the ReLU activation, culminating in a SoftMax activation. Moreover, each downblock layer establishes a skip connection to its corresponding upblock layer, ensuring seamless information flow. The kernel count within each convolutional layer of a given downblock remains consistent and is doubled progressively up to downblock-4. However, in downblock-5 and downblock-6, the kernel count in the second convolutional layer decreases by one-fourth and half of downblock-4, respectively. Conversely, upblock-6 through upblock-4 maintain uniform kernel counts, whereas those in upblock-3 through upblock-1 are relative to their preceding upblock counterparts, as depicted in Table 1. Additionally, our proposed mA-UNet middle block encompasses a max-pooling layer flanked by two 3×3 convolutional networks featuring ReLU activation, and a dropout layer, interconnected via a 2×2 convolutional transpose with consistent padding dimensions.

Again, the architecture is composed of a contraction (down-sampling) path and an expansive (up-sampling) path, which are connected to facilitate precise feature fusion across multiple resolution levels. Given an input tensor $X\in R^{H\times W\times C}$, each convolutional block in the encoder applies two successive convolution operations with 3×3 kernels, followed by nonlinear activation and dropout regularization. Mathematically, the convolution operation at layer *l* can be expressed as

$$F_{i,j,k}^{\left(l\right)}=\sigma \;\left(\sum_{m,n,c}W_{m,n,c,k}^{\left(l\right)}\;F_{i+m,j+n,c}^{\left(l-1\right)}+b_{k}^{\left(l\right)}\right)$$

where $W^{\left(l\right)}$ and $b^{\left(l\right)}$ denote the learnable kernel weights and biases, respectively, and σ(·) represents the ReLU activation function. Spatial down-sampling is subsequently performed using max-pooling layers with a stride of 2, which can be defined as

$$P_{i,j,k}^{\left(l\right)}=\max_{(m,n)\in \Omega }F_{2i+m,\;2j+n,\;k}^{\left(l\right)}$$

This reduces the spatial resolution while progressively increasing the representational capacity of the feature maps. At the bottleneck, the network attains highly abstract semantic representations with the largest receptive field. In the expansive path, spatial resolution is restored through transposed convolution operations, formulated as

$$U^{\left(l\right)}=F^{(l-1)*}W_{\mathrm{up}}^{\left(l\right)}$$

where ∗ denotes the transposed convolution with stride 2 that doubles the spatial dimensions. Each up-sampled feature map is concatenated with the corresponding encoder feature map via skip connections, enabling the integration of low-level spatial information with high-level semantic features. Subsequent convolutional layers refine these fused representations, while dropout layers enhance generalization. Finally, a 1×1 convolution followed by a softmax activation maps the refined features into the target label space, yielding a dense pixel-wise probability distribution over the predefined classes. Collectively, this architectural design allows mA-UNet to achieve robust multiscale feature learning, stable gradient propagation, and improved segmentation performance.

### 2.3. Hyperparameter Tuning

The influence of hyperparameters on the behavior of training algorithms is noteworthy, given their potential to directly impact the performance of the resultant model. The quest for optimal hyperparameters has traditionally involved manual determination, with researchers relying heavily on prior experience in training machine learning algorithms. However, this practice presents drawbacks, as the optimal hyperparameter configuration for one problem may not necessarily translate to another, owing to variations across datasets and image categories. Consequently, establishing hyperparameter values solely based on past experiences poses a formidable challenge [38].

In our endeavor to enhance model efficiency concerning dropout and kernel count per layer, we leverage the auto Keras framework [39]. To navigate this complex landscape, the framework optimizes an acquisition function—typically Expected Improvement (EI) to identify which structural modifications are most likely to minimize the loss function L(θ) or maximize accuracy metrics. When the searcher selects a specific morph operation, such as increasing the filter count in a convolutional layer or inserting a skip connection, it initializes the new parameters such that the output of the layer f(x) remains numerically consistent with the pre-morphed state. This framework employs Bayesian optimization techniques [40], traversing the search space to identify promising operations, thus facilitating an efficient neural architecture search tailored to hyperparameter tuning. Additionally, it employs an edit distance neural network kernel to quantify the transition between different neural network configurations. Subsequent to tuning, we determine the optimal number of kernels per layer, as shown in Table 1, where each convolutional layer’s specifications, including feature height, width, and kernel count, are provided in parentheses. Moreover, when gradient descent approaches a minimum of the cost function, parameter values may oscillate around it, necessitating further optimization. To address this, we implement a learning rate scheduler that progressively reduces the learning rate after specified epochs, thereby mitigating fluctuations and improving the efficiency of gradient computation.

### 2.4. Loss Function

Binary Cross Entropy (BCE) [41] is a widely used loss function in image segmentation tasks for its numerous advantages, including suitability, pixel-wise accuracy, effectiveness with imbalanced data distributions, probabilistic interpretability, and differentiability. In our proposed framework, adopting BCE loss is significant, as it is expected to improve model performance. BCE is defined as the difference between each predicted probability and the corresponding actual class label, typically binary (0 for the negative class and 1 for the positive class). Subsequently, a score is computed that reflects the extent to which the predicted probabilities diverge from the expected values, thereby quantifying the proximity or disparity between the estimated and true values. The optimization process, as delineated in Equation (5), encapsulates the refined solution.

(5) $$CE=-gt_{1}\log\left(f\left(sr_{1}\right)\right)-\left(1-gt_{1}\right)\log\left(1-f\left(sr_{1}\right)\right)$$

### 2.5. Evaluation Metrics

Using a binary segmentation approach, our results are either positive, indicating the presence of a nucleus, or negative, indicating the background. In the segmentation process, correctly identifying a nucleus results in a true positive (tp), whereas accurately labeling background pixels yields a true negative (tn). Instances where the background is wrongly classified as the nucleus are termed false positives (fp), whereas false negatives (fn) arise when background pixels are incorrectly identified as nucleus pixels. Various metrics can be employed to assess segmentation efficiency, and we are particularly interested in the accuracy, defined as the proportion of correctly labeled pixels among the total. For the evaluation of our experiment, precision, recall, F1 [42], and MeanIoU are being used. Every term and its derivation are defined and illustrated by Equations (6)–(8).

(6) $$precision=\frac{tp}{tp+fp}$$

(7) $$recall=\frac{tp}{tp+fn}$$

(8) $$f1-score=2\times \frac{precision\times recall}{precision+recall}$$

For the segmentation evaluation, the MeanIoU score is a commonly used performance metric. The MeanIoU measure is given in Equation (9).

(9) $$IoU=\frac{tp}{tp+fp+fn}$$

### 2.6. mA-UNET Architecture

**Figure 2:**
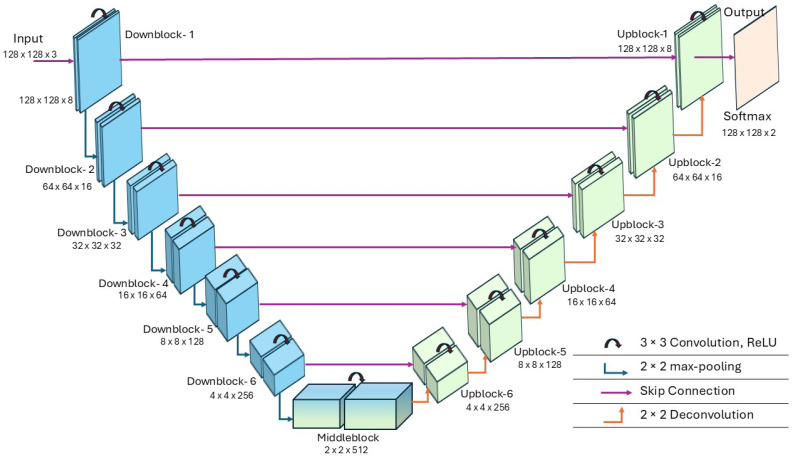
The proposed mA-UNET model architecture.

The mA-UNet architecture comprises an encoder network (downblock-1 to downblock-6) and a decoder network (upblock-1 to upblock-6), as illustrated in Figure 2. The encoder progressively reduces the spatial dimensions of the input image while increasing the number of feature channels, enabling it to capture high-level semantic information. Starting with a 128×128×3 input image, six consecutive downsampling blocks apply 3×3 convolutions with ReLU activation, followed by 2×2 max-pooling, which sequentially halves the spatial dimensions while increasing the feature depth from 8 to 256 channels. The bottleneck layer at the deepest part of the network handles feature maps with dimensions of 2×2×512. The decoder then upsamples these feature maps and combines them with corresponding feature maps from the encoder via skip connections to recover spatial details. These skip connections ensure that information from earlier layers is preserved, enhancing the segmentation accuracy. The final layer applies a Softmax activation function to produce the output.

**Figure 3:**
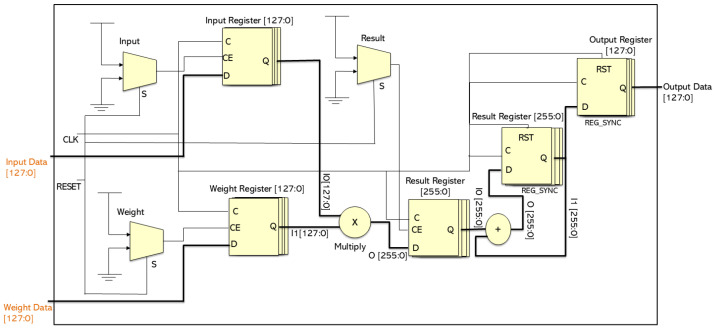
The hardware implementation for the convolutional unit of the proposed mA-UNET model.

Figure 3 depicts the hardware implementation of a single convolutional unit, which processes input data and weights, performs convolution through multiplication and accumulation, and outputs the result. The design is optimized for pipelined operation, resulting in increased throughput and efficiency. This convolutional block is combined with others to form a complete convolutional layer. The input and weight registers store the necessary data and weights for the convolution process, and the operation is synchronized using the clock (clk) and reset (reset) signals. Multiplexers select between two data sets—inputs and weights—to facilitate the selection of specific data segments or to manage data flow during different stages of the pipeline. Selection lines control the data path through the pipeline. The multiplier computes the product of the selected input and weight data, and the intermediate results are stored in a temporary register that accommodates the increased bit width resulting from the multiplication. An adder accumulates the intermediate results to generate the final convolution output, which is saved in another register. The final output is stored in an output register and is ready for transfer out of the module. The use of registers at various stages indicates a pipelined architecture, where multiple operations can be executed concurrently at different stages, thereby increasing throughput.

Each convolutional block accepts input and weight parameters, along with biases that modify the output after convolution. The parameters for each block are supplied with varying bit widths, corresponding to different phases of the processing pipeline, which likely align with different network layers. The blocks are arranged progressively from downblock-1 to downblock-6, with decreasing data and weight sizes, indicating a downsampling process that gradually reduces the spatial dimensions or complexity of the input. The middle block, positioned at the lowest resolution, performs crucial feature extraction. The upsampling blocks mirror the downsampling blocks, but with parameters set in reverse to increase data size, signifying an upsampling process. The growing data and weight sizes in the upsampling blocks suggest that these layers refine feature resolution while integrating learned features from deeper layers. The detailed bit-level data management optimizes hardware resource utilization, which is critical for performance-sensitive applications.

## 3. Implementation and Experimental Results

### 3.1. Dataset

**Figure 4:**
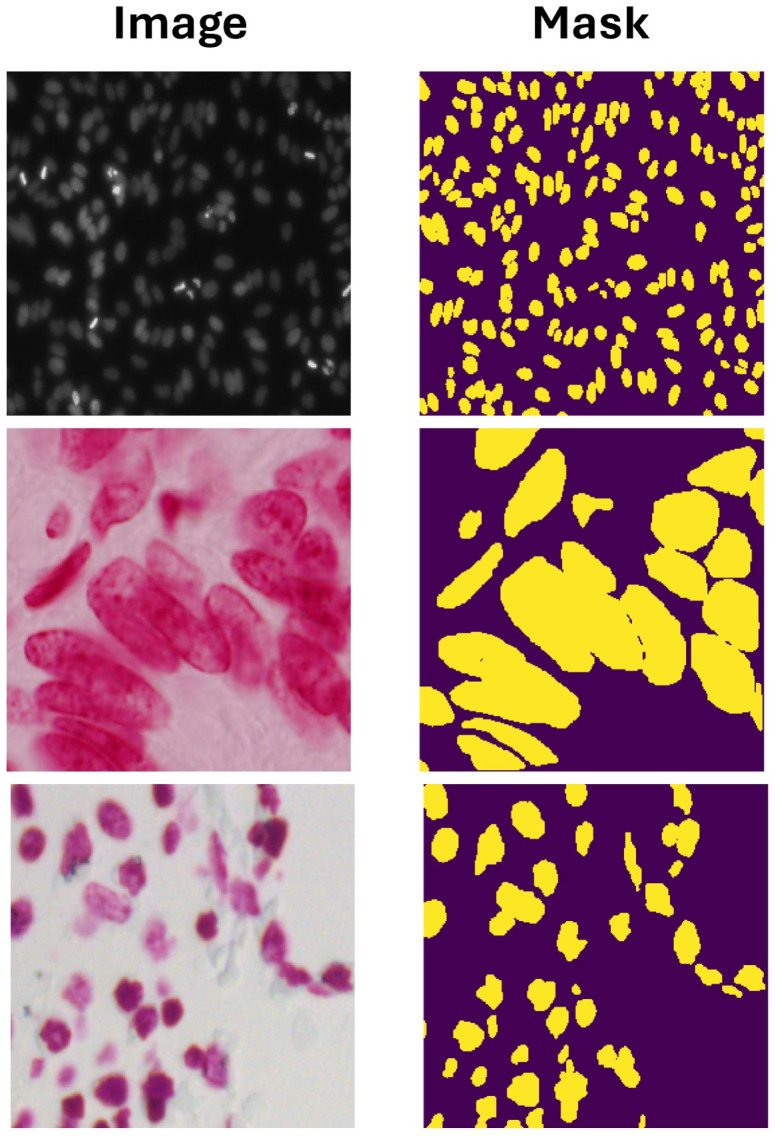
Examples from the 2018 Data Science Bowl Dataset, where the first column indicates the image and the second column indicates the mask.

In our experimental setup, we utilized the 2018 Data Science Bowl [43], curated initially for a three-month competition. During this competition period, participants were granted access to both the training set, which included target masks, and the test set, in which target masks were withheld. As shown in Figure 4, examples of the dataset are visualized in RGB format using Matplotlib (3.10). Comprising 841 2D images, this dataset encompasses a total of 37,333 manually annotated nuclei originating from over 30 experiments conducted across diverse samples, cell lines, microscopy instruments, imaging conditions, operators, research facilities, and staining protocols. These annotations were produced through a collaborative workflow undertaken by a team of expert biologists; hence, they are termed ‘target masks’ rather than ground truth, reflecting the single expert annotation process followed by collective review. The images were generously contributed by researchers worldwide under a Creative Commons 0 license, thereby falling into the public domain, and the team’s annotations are similarly accessible. This dataset is openly available within the Broad Bioimage Benchmark Collection, identified by accession number BBBC038.

### 3.2. Training

**Figure 5:**
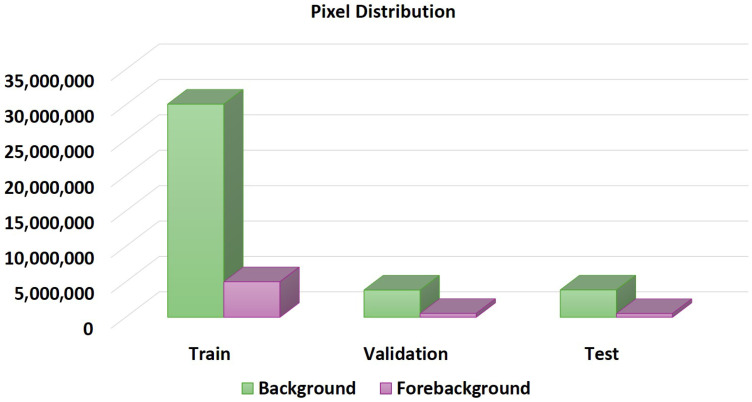
Pixel distribution of the 2018 Data Science Bowl Dataset.

The entirety of our training framework is constructed utilizing the Keras Library, a tool renowned for its efficiency in deep learning applications. In preparation for training, we partitioned the dataset into three distinct subsets after the pre-processing steps: training, validation, and testing, ensuring a robust evaluation process. The random seed was set to 42 to make pseudo-random processes reproducible, ensuring that the same random sequence is generated each time the code runs. Given the heterogeneous nature of the images within the dataset, characterized by varying shapes and complexities, managing them posed a significant challenge. However, the integration of the ADA method [35] facilitated seamless image pre-processing, preserving essential information while minimizing potential losses. Using this approach, we transformed the initial 536 training images into an ensemble of 2628 patch images and 55 validation images into an ensemble of 267 patch images, each standardized to 128×128. Notably, in determining the parameter alpha referenced in Equation (4), a value exceeding 9 was selected, signifying a threshold of >9 foreground pixels per image, thereby ensuring the inclusion of images with a minimum of 10% foreground coverage. The distribution of pixels across the dataset is analyzed on a pixel-wise basis, and Figure 5 clearly shows the imbalance in the dataset.

The training process used the Adam optimizer combined with the Binary Cross Entropy Loss function, using a batch size of 64, epochs of 500, and an initial learning rate set to 0.0003. Additionally, we incorporated the Keras learning rate scheduler, a dynamic mechanism that adjusts the learning rate at the onset of each epoch. This scheduler updates the learning rate according to a predefined schedule function, accounting for the current epoch and the current learning rate. The function implements a performance-aware learning rate decay strategy that adjusts the optimization step size during training. Specifically, the function computes the learning rate as a function of the current training epoch, where a multiplicative decay factor is applied at regular intervals determined by a fraction of the total number of training epochs. By defining a decay coefficient of 0.5 and an epoch interval equal to one-eighth of the total training duration, the learning rate is reduced by half each time the epoch index surpasses a predefined threshold. Moreover, the use of a floor operation ensures that the decay is applied in discrete steps rather than continuously, which prevents excessively frequent fluctuations and promotes smoother optimization dynamics. Consequently, this scheduling mechanism enables larger learning rates during the early stages of training to encourage rapid exploration of the parameter space, while progressively decreasing the learning rate in later epochs to refine the solution and mitigate oscillations around local optima, enhancing overall training stability and generalization performance.

To prevent overfitting, a dropout rate of 20% was applied within the hidden layers of the network architecture. Following each training iteration, the model’s performance was evaluated using validation images to compute metrics such as F-measure and MeanIoU. This iterative process continued throughout training, culminating in the preservation of the model with the highest F-measure and MeanIoU values. In addition, we used an early stopping callback function where we set patience to 50, which means the number of epochs with no improvement after which training will be halted. We also set weight decay to 0.0001 and provided the learning rate as a parameter for the Adam optimizer. Again, a custom callback class was integrated to record training and validation accuracy metrics, preserve the best model weights, and visualize randomly sampled masks from the validation dataset, enabling a comprehensive assessment of the model’s performance.

### 3.3. Experimental Results Analysis

This section examines our comprehensive performance analysis, which serves to underscore the superiority of our approach over alternative models. The outcomes derived from the utilization of test images are presented in Table 2, representing the models in the first column, with our model’s performance highlighted in bold in the final row. The tabulated data distinctly showcases the enhanced performance of our proposed methodology, as evidenced by an across-the-board improvement across all four evaluation metrics. Notably, the closest performance to our proposed framework is observed in UNet++, attaining a MIoU score of 0.926. Additionally, seUNet-Trans-L notably achieves a precision score of 0.960, closely resembling our model’s performance, although falling short in other evaluative aspects. Conversely, our method surpasses all previously proposed frameworks in terms of recall and f-1 score, further underscoring its superior performance and efficiency.

**Figure 6:**
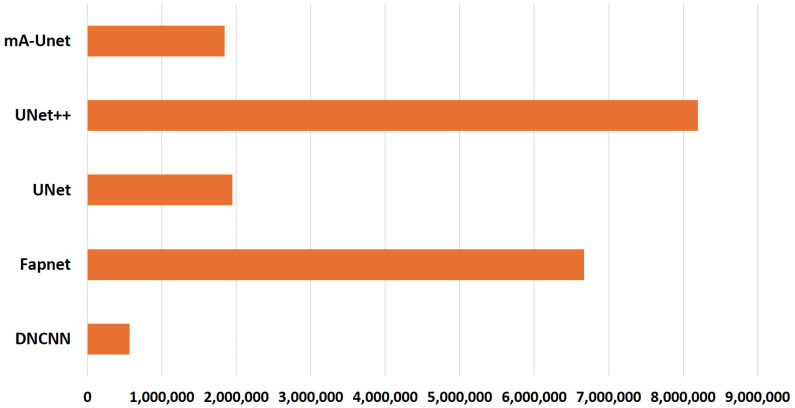
Parameter comparison of mA-UNet with other state-of-the-art models.

Illustrated in Figure 6 is a comparison of parameter sizes across select lightweight state-of-the-art models. We used the TensorFlow summary function to compute and report the parameter counts for each model. This utility generates a comprehensive tabular overview that details the number of parameters associated with every individual layer, while also explicitly distinguishing between trainable and non-trainable parameters, as well as reporting the aggregate parameter count for the entire model. Consequently, this approach ensures a transparent, standardized, and reproducible assessment of model complexity across different architectures. This visualization highlights a notable trend: our model has fewer parameters than most of its counterparts while achieving superior performance compared with other state-of-the-art models. In particular, despite DNCNN having fewer parameters than mA-UNet, its performance significantly lags behind that of the proposed network.

**Figure 7:**
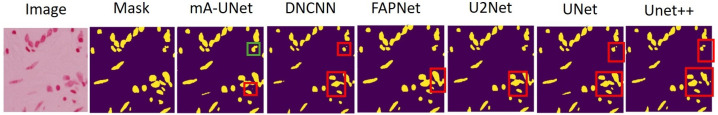
Example taken from test set for qualitative analysis where six predictions are provided along with an original mask and the feature image. All the figures are plotted in RGB mode, where the yellow color indicates the positive class (nucleus) prediction. The green box indicates the model’s ability to capture small details and the red box indicates the shortcomings of perfectly predicting the area.

From our testing dataset, we randomly selected a single prediction, represented in Figure 7, which showcases six distinct predictions alongside the original mask and feature image. Within this visual representation, we have identified two illustrative scenarios, highlighted by red boxes, representing the challenging circumstances encountered during nucleus segmentation. Our analysis reveals a common trend among most models, whereby the nucleus is accurately predicted with some minor oversights in capturing finer details. Particularly, in the first scenario delineated by a green box, mA-UNet, FAPNet, and U2Net successfully predict a small area. Conversely, in the second scenario, nearly all models fall short of perfectly predicting the nucleus, Although mA-UNet achieves remarkable proximity. In summation, mA-UNet demonstrates a superior ability to predict nuclei with greater precision compared to other state-of-the-art models, without becoming biased toward background pixels.

### 3.4. Hardware Resources Utilization

The proposed mA-UNet accelerator was synthesized and implemented using Xilinx Vivado Design Suite v2022.2, targeting the Zynq UltraScale+ platform. All reported results correspond to post-placement and routing (post-PAR) timing analysis. The design employs fixed-point arithmetic to reduce hardware complexity and power consumption, while maintaining segmentation accuracy. Table 3 provides an overview of the hardware resource utilization for our method. The implementation demonstrates efficient use of the available resources. Only 1145 of the 230,400 available Slice LUTs were utilized, representing a low usage rate of 0.5%. Similarly, 1268 of the 460,800 available slice registers were used, resulting in an even lower usage rate of 0.28%. In terms of specialized resources, the network required just one of the 544 available BUFs (0.18%) and 96 of the 1728 available DSPs, reflecting a utilization rate of 5.56%. Notably, no Block RAM resources were required, demonstrating the design’s minimal memory requirements. Additionally, 130 bonded IOBs were used out of a possible 464, yielding a relatively higher utilization rate of 28.2%. These numbers highlight the network’s ability to handle complex computations efficiently while using minimal hardware space, demonstrating the design’s scalability and effectiveness on advanced FPGA platforms. Table 4 summarizes the Performance metrics of the proposed mA-UNet FPGA implementation, including post-placement-and-routing timing results, power consumption, clock frequency, and real-time processing capability. The design operates at 132.08 MHz using fixed-point arithmetic and processes 128 × 128 inputs in a fully streaming manner, achieving high throughput with low latency and modest power consumption.

## 4. Conclusions

This paper presents an innovative methodology, termed mA-UNet, which utilizes the robust feature extraction capabilities of CNN with the contextual comprehension of small foreground–background pixels, thereby advancing microscopy image segmentation. mA-UNet employs a hybrid architecture that integrates the fully convolutional UNet with the ADA data augmentation algorithm [35]. Central to this integration is a specially designed bridge layer that sequentially transmits rich feature maps from UNet. The proposed approach yields results competitive with other leading nucleus segmentation methodologies. As demonstrated in Table 2, our models exhibit superior performance in terms of both F-measure and MIoU when benchmarked against state-of-the-art models using the 2018 Data Science Bowl dataset [43]. Furthermore, the proposed framework incorporates the ADA augmentation algorithm [35], which not only enhances performance but also mitigates data loss by accommodating input frames of varying dimensions. The mA-UNet model is implemented in VHDL on the Zynq UltraScale+ FPGA, demonstrating the design’s efficiency and scalability on advanced FPGA platforms.

The encouraging findings outlined in this investigation set the stage for the broader deployment of our proposed model across diverse tasks. Future endeavors will focus on tailoring our approach to meet specific application requirements. Our primary objective is to refine our methodology to achieve expedited inference times, thereby enhancing its practical utility. To surpass our current performance benchmarks, we aim to incorporate a temporal filter to improve classification accuracy. Additionally, we plan to explore the integration of advanced methodologies, such as the Swin Transformer, to further enhance the effectiveness of our model. These avenues of inquiry are expected to make substantial contributions not only to the domain of medical image analysis but also to adjacent fields.

## Figures and Tables

**Table 1:** The layer construction of mA-UNet model.

Layers	Conv2D1	Conv2D2	Padding	Activation Function
**Input **	(128, 128, 3)	-	-	-
**downblock-1**	(128, 128, 8)	(128, 128, 8)	Same	ReLU
**downblock-2**	(64, 64, 16)	(64, 64, 16)	Same	ReLU
**downblock-3**	(32, 32, 32)	(32, 32, 32)	Same	ReLU
**downblock-4**	(16, 16, 64)	(16, 16, 64)	Same	ReLU
**downblock-5**	(8, 8, 128)	(8, 8, 16)	Same	ReLU
**downblock-6**	(4, 4, 256)	(4, 4, 32)	Same	ReLU
**middleblock**	(2, 2, 512)	(2, 2, 64)	Same	ReLU
**upblock-6**	(4, 4, 256)	(4, 4, 32)	Same	ReLU
**upblock-5**	(8, 8, 128)	(8, 8, 16)	Same	ReLU
**upblock-4**	(16, 16, 64)	(16, 16, 64)	Same	ReLU
**upblock-3**	(32, 32, 32)	(32, 32, 32)	Same	ReLU
**upblock-2**	(64, 64, 16)	(64, 64, 16)	Same	ReLU
**upblock-1**	(128, 128, 8)	(128, 128, 8)	Same	ReLU
**Output**	(128, 128, 2)	-	-	Softmax

**Table 2:** The experimental results on the 2018 Data Science Bowl Dataset comparing the performance of the proposed mA-UNet with the state-of-the-art models.

Models	MIoU	Precision	Recall	F-1 Score
DNCNN [44]	0.897	0.938	0.926	0.932
FAPNET [45]	0.887	0.925	0.945	0.935
Unet [24]	0.910	0.938	0.943	0.940
UNet++ [28]	0.926	0.928	0.873	0.899
U2Net [46]	0.886	0.502	0.602	0.547
seUNet-Trans-L [47]	0.860	0.96	0.894	0.926
seUNet-Trans-M [47]	0.860	0.947	0.911	0.929
seUNet-Trans-S [47]	0.840	0.95	0.884	0.916
FANet [32]	0.857	0.922	0.919	0.920
DoubleU-Net [31]	0.841	0.841	0.641	0.727
DS-TransUNet-L [33]	0.861	0.912	0.938	0.924
mA-Unet	**0.955 **	**0.966**	**0.970**	**0.978**

**Table 3:** Hardware Resources Utilization Result.

Logic Utilizing	Available	Used	Utilization
**Number of Slice LUTs**	230,400	1145	0.5%
**Number of FFs**	460,800	1268	0.28%
**Number of BUFs**	544	1	0.18%
**Number of DSPs**	1728	96	5.56%
**Number of Block RAM**	312	0	0%
**Bonded IOB**	464	130	28.2%

**Table 4:** Synthesis and Execution Parameters of the mA-UNet FPGA Implementation.

Parameter	Value
**Target FPGA**	Zynq UltraScale+
**Toolchain**	Xilinx Vivado v2022.2
**Quantization Scheme**	Fixed-point
**Clock Frequency**	132.08 MHz
**Power Consumption**	0.848 W
**Latency**	3.88 μs
**Throughput**	8060.8 frames/s

## Data Availability

Data are contained within the article.
